# COVID-19 Diagnosis Using an Enhanced Inception-ResNetV2 Deep Learning Model in CXR Images

**DOI:** 10.1155/2021/6658058

**Published:** 2021-06-03

**Authors:** Madallah Alruwaili, Abdulaziz Shehab, Sameh Abd El-Ghany

**Affiliations:** ^1^Department of Computer Engineering and Networks, College of Computer and Information Sciences, Jouf University, Sakaka, Saudi Arabia; ^2^Department of Information Systems, College of Computer and Information Sciences, Jouf University, Sakaka, Saudi Arabia; ^3^Department of Information Systems, Faculty of Computers and Information, Mansoura University, Mansoura 35516, Egypt

## Abstract

The COVID-19 pandemic has a significant negative effect on people's health, as well as on the world's economy. Polymerase chain reaction (PCR) is one of the main tests used to detect COVID-19 infection. However, it is expensive, time-consuming, and lacks sufficient accuracy. In recent years, convolutional neural networks have grabbed many researchers' attention in the machine learning field, due to its high diagnosis accuracy, especially the medical image recognition. Many architectures such as Inception, ResNet, DenseNet, and VGG16 have been proposed and gained an excellent performance at a low computational cost. Moreover, in a way to accelerate the training of these traditional architectures, residual connections are combined with inception architecture. Therefore, many hybrid architectures such as Inception-ResNetV2 are further introduced. This paper proposes an enhanced Inception-ResNetV2 deep learning model that can diagnose chest X-ray (CXR) scans with high accuracy. Besides, a Grad-CAM algorithm is used to enhance the visualization of the infected regions of the lungs in CXR images. Compared with state-of-the-art methods, our proposed paper proves superiority in terms of accuracy, recall, precision, and *F*1-measure.

## 1. Introduction

With the continuation of the COVID-19 pandemic, the number of infected people increases daily. The number of deaths is rising, especially for elderly and ill people. Consequently, there is an urgent need to discover new ways to diagnose and identify this virus early to minimize its effects. The PCR test is considered as the fundamental screening and the golden standard technique for COVID-19 diagnosis. However, one of its limitations, as reported by the clinical experience, is having a low positive rate in the early stage of infection [1, 2], and it takes 4–6 hours to get the result, which is deemed to be a long period with the fast spread rate of COVID-19 [3]. Therefore, it was recommended to rely on tests taken by chest X-ray (CXR) images and computed tomography (CT) scan as an alternative method for PCR test and as one of the early diagnostic methods [4–6]. However, the challenge of such CXR images or CT scans demands both radiologists and considerable time to visually examine each CXR image and extract important findings. In addition, it has become difficult for radiologists, especially the novice, to figure out these minor variations with the naked eye, due to similar trends and overlaps of infectious and inflammatory lung diseases. Dependently, an automated Computer-Assisted Diagnosis (CAD) system is urgently required to save time and develop intelligent solutions to help radiologists get an accurate diagnosis of COVID-19. CAD systems play a significant role in the medical field offering early diagnosis of disease progression in cost-effective and impartial terms in relation to human interventions. Motivated by the urgent need to develop solutions to aid in facing the COVID-19 pandemic, this paper proposes an expert comprehensive CAD system for early diagnosis of COVID-19 cases depending on deep learning (DL) techniques for CXR image analysis. Recently, DL has become the main technology of increasing artificial intelligence in automatic diagnosis of lung disease detection, through medical imaging analysis. It is a leading technique in radiology diagnosis, which has produced the solutions required for disentangling awareness in lung pathology. The proposed model will help clinicians in a way that discloses confirmed cases, either pressing or simple to be minimized. The model relies on particular lung regions to predict and diagnose whether the patient has COVID-19. The output can then be represented in a heatmap-like plot using the class activation map (CAM) algorithm locating the affected lung areas. The output of this system could then be helpful to be used by medical professionals, especially those in the limited test kit areas or those who are struck by an unexpected increase in suspected cases. Moreover, the system could rapidly differentiate between COVID-19-infected patients and those who are infected with common pneumonia or another disease. The proposed work offers great potential by reducing pressure on front-line radiologists, improving early diagnosis and treatment, and accordingly controlling the epidemic. This model may be used to support radiologists at this stage and to resolve this situation. Besides, it reduces the pressure on laboratories that make diagnoses based on an analysis of throat and pharyngeal swabs.

The contributions of this paper are summarized as follows:

- Building a system that can diagnose CXR scans with high accuracy to improve the early diagnosis of the disease and thus contribute to controlling the epidemic and to reduce the time required to diagnose the COVID-19 cases
- Building an ensemble of DL models in a new framework and proposing an enhanced DL model that utilizes a new transfer learning algorithm with the ability to overcome the problem of overfitting that makes it more efficient in real time
- Constructing up a concatenating Inception-ResNetV2 model that overcomes the performance of other related studies
- Localizing the disease by using a Grad-CAM algorithm that visualizes the infected areas of the lungs in CXR images

The rest of this paper is organized as follows.  Section 2 shows a literature review related to the COVID-19 diagnosis system. In Section 3, there is a detailed description of the model architecture and materials. The implementation and evaluation are presented and discussed in Section 4, while Section 5 presents conclusions and future work.

## 2. Literature Review

Many researchers have employed various techniques of artificial intelligence to address the COVID-19 pandemic; for example, Imran et al. [7] presented a system for diagnosis of COVID-19 patients by recognizing the coughing sound. The proposed system consists of two parts: the first is cough detection, whereas the second is COVID-19-diagnosis-based predetected cough sound. There are many recent studies that use machine learning techniques for the diagnostic of CT and CXR scans as an alternative to PCR test. For example, Farooq and Hafeez [8] presented COVID-ResNet. Their work proposed a ResNet deep learning approach for multiclass classification of normal, bacterial, viral, and COVID-19 classes. Their system achieved accuracy of 96.23% on all classes. Hassantabar et al. [9] presented in their research work three types of DNN and CNN methods, two of which were to diagnose the lungs of patients with X-ray images infected with COVID-19 and the last one to detect the infected regions in the lung. The first method that was used to diagnose COVID-19 is based on the use of the fractal feature of images as input to the CNN model for binary classification to COVID-19 and non-COVID-19 cases. The classification accuracy of their model was 93.2%. However, their model detects infected regions with 83.84% accuracy.

Zhang et al. [10] proposed a deep learning model for COVID-19 detection based on chest X-ray images. Their model trained on 100 chest X-ray images for COVID-19 and 1431 images for pneumonia cases and got overall 96% sensitivity and 70.65% specificity.

Qi et al. [11] analyzed 57 positive cases of COVID-19 by using the chi-square technique. They tried to approve the importance of demographic data, clinical data, and chest CT scans in diagnosis of the COVID-19 patients. They concluded that CT features and dynamic observation play an essential role in detecting and diagnosing COVID-19 cases. Wang et al. [12] proposed a deep convolutional neural network (CNN), which is called COVID-Net, to diagnose the COVID-19 cases based on CXR images. Their proposed COVID-Net can diagnose three different types, which are normal, pneumonia, and COVID-19. Their network can get accuracy by achieving 93.3% test accuracy. Wang et al. [13] used the Inception network model for diagnosing COVID-19. Their network contains three essential phases, which are preprocessing, feature extraction, and classification. By using CT imaging feature extraction, their network achieved 89.5% accuracy [14]. Torman et al. proposed a deep learning model called CapsNet for the detection of COVID-19 by using CXR images. The model gives an accuracy of 97.24% for binary classification and 84.22% accuracy for multiclass classification.

Song et al. [15] developed a DL network, which is called DeepPneumonia, to diagnose COVID-19 cases depending on analyzing CT scans. Their proposed system was built on the ResNet50 using transfer learning technology. It could localize the essential lesion characteristics, especially ground-glass opacity (GGO). Their system achieved an average area under the curve (AUC) of 99% and sensitivity score of 93%. Besides, it reached an average AUC of 95% and sensitivity of 96% for bacterial pneumonia-infected cases. Xu et al. [16] proposed a fully automated COVID-19 diagnosis based on a 3D deep learning network using chest CT scans. Their proposed system consists of four basic stages, which are preprocessing, candidate region segmentation, classification for each candidate region, and overall infection probability. Bukhari et al. [17] employed ResNet50 for COVID-19 detection using CXR images. They tried to differentiate four types of classes, which are healthy normal, bacterial pneumonia, viral pneumonia, and COVID-19 cases. They achieved an average accuracy of 98.18 % and *F*1-score of 98.19%. Khan et al. [18] proposed a model named CoroNet to identify COVID-19 in X-ray and CT scans utilizing a pretrained Xception convolution network. Two tests were conducted to validate their model. For the four classes (viral pneumonia, COVID-19, bacterial pneumonia, and normal), the first experiment attained an accuracy of 89.6 %, while the second experiment for three classes (normal, COVID-19, and pneumonia) obtained a total accuracy of 95%.

A recent COVIDX-Net model to help radiologists in identifying and diagnosing COVID-19 in CXR images was developed by Hemdan et al. [19]. They compared seven performances of seven pretrained DL networks; they are the InceptionV3, MobileNetV2, VGG19, DenseNet201, Inception-ResNetV2, ResNetV2, and Xception model. Based on their experiments, the VGG model achieved the highest accuracy of 90%. Sethy and Behera [20] introduced a hybrid approach that utilizes deep learning for feature extraction and support vector machine (SVM) for detecting patients contaminated with COVID-19 by using CXR images. The deep features of the CNN layer model are extracted and fed into SVM for the classification process. Their approach is useful for a physician to identify cases, pneumonia patients, and healthy persons among COVID-19. By using the pretrained 13 distinct CNN models, the SVM provided the best results on the deep features of the ResNet50 model. Ouchicha et al. [21] proposed a model named CVDNet to diagnose the COVID-19 cases. This model focuses on a residual neural network and employed local and global features of chest X-ray images by utilizing two parallel layers with various kernel sizes. Their proposed CVDNet has achieved an average accuracy of 97.20% for detecting COVID-19 cases. These experiments obtained correct responses to the COVID-19 pandemic. However, they have certain drawbacks to remember. In the best case, they used tiny databases of less than 400 COVID-19 X-ray images.

## 3. Materials and Methods

### 3.1. Dataset Description

In this study, the samples of CXR images covered three main classes labelled as confirmed COVID-19, viral pneumonia, and normal (healthy) cases. All images are of size 299 × 299. COVID-19 class contains the identified COVID-19-positive cases confirmed by the CXR image and specialists. Viral pneumonia class contains the patient's patches of pneumonia infection. Finally, the normal class includes the radiology images of various cases, which are neutral and have no lung infection. The CXR images are attained from COVID-19 Radiography Dataset [22].  Table 1 describes the distribution of training and testing sets employed in the experiment for 80–20 training-testing set. It contains a total amount of 2905 CXR images, which are distributed into 219 COVID-19 images, 1345 viral pneumonia images, and 1341 for normal category.

Besides, a team of researchers have created a new release of the dataset [22]. It contains four classes: COVID-19, normal, lung opacity (non-COVID-19 lung infection), and viral pneumonia. The lung opacity class is ignored in our experiments to preserve the symmetry with old dataset in terms of number of classes. COVID-19 class has increased to 3616 CXR images along with 10,192 images in the normal class, 6012 lung opacity, and 1345 viral images.  Table 2 presents the distribution of training, validation, and testing sets used in the experiment.

### 3.2. Model Architecture and Model Training

**Figure 1:**
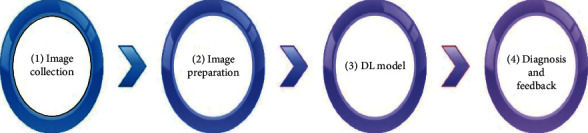
System overview.

Nowadays, CNNs are proving their real success in the classification task. The detailed proposed model, which will be discussed in the next section, aims for an automated system capable of processing CXR images to detect COVID-19 disease. The system overview is described as shown in Figure 1. (1) Images were collected from COVID-19 Radiography Database. (2) The images are preprocessed via artifact removal, resizing, contrast handling, and normalization, which will be explained later. (3) Furthermore, the images are classified with an ensemble of DL models and we focused on our core model named the Inception-ResNetV2 model. (4) Disease diagnosis and feedback are provided using Grad-CAM algorithm.

**Figure 2:**
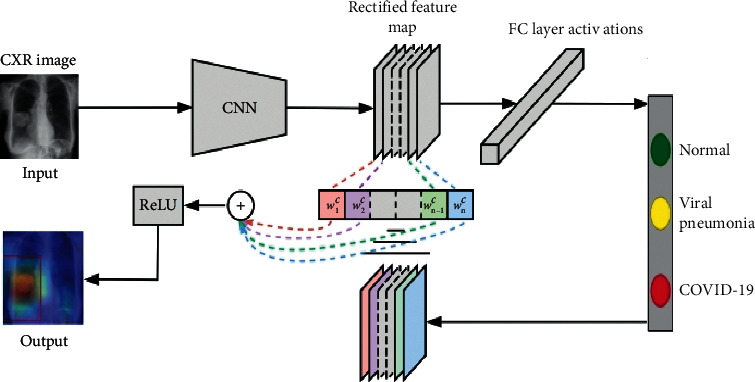
The overall model architecture.

Initially, the process of image collection is accomplished, followed by image preparation and DL model, and finally ends with diagnosis and feedback. Occasionally, data preparation is one of the most important steps, since preparing the collected data in an efficient manner leads to accurate results. It contains operations like equalizing the number of images in each class, simple filtering, denoising, etc. Subsequently, the utilized dataset can be divided into two sets: training and testing sets. Through the training process, many tuning experiments are done to get the optimized network parameters. More model learning yields more accurate results in classification output. Dependently, the model is then tested on the remaining invisible images (testing set) and the DL network, after a number of experiments become converged and deployed. More details about the proposed system architecture/network are given in Figure 2.

Initially, the dataset image's pixel representations are presented to the CNN, in which layers are interconnected together in a multilayer architecture. Then, the network is responsible for turning the input visual stimulus into nonlocal signals, which becomes more complex as it passes through many succeeding layers. Initial layers are capable of capturing simple features like edges, corners, intensity values, and texture, while complex features are gradually formed by the abstraction higher layers. Inception-ResNetV2 is our main/core DL model due to its superiority in the experimental results. However, there are also many other DL models, which are performed and documented. The performance of DL models is highly based on the number of images used to train the model. Therefore, it requires a large training dataset to extract temporal and spatial features. However, even if the dataset is not large enough, the DL model can be employed through using what is known as transfer learning approach.

Transfer learning has become one of the popular considerable methods utilized for detecting/classifying COVID-19 cases. It is mainly based on how to reuse the expertise or knowledge gained from one mission into another. It is an efficient method, especially if the intended model has a limited dataset.

In transfer learning, the feature extracted (learning) from a DL network is transferred to solve related problems with small dataset and which cannot be implemented from scratch [23]. ImageNet [24] is one of the popular large datasets used in the medical domain. The choice of the suitable DL model is highly dependent on its ability to extract the features related to the domain. During the feature extraction, the pretrained model can capture the new features from the dataset. Thereafter, a parameter tuning process, by updating and reconstructing the model architecture, is necessary to optimize the model performance in the new applied domain. In this way, the pretrained model overcomes the small dataset issues; consequently, the computational cost is significantly decreased. Hence, the transfer learning approach is applied to take advantage of the generalizability of DL models, especially Inception-ResNetV2 model. In this study, the models are pretrained on ImageNet to capture the initial parameters, speed up the training convergence, and improve the classification accuracy.

#### 3.2.1. Data Preprocessing

In this study, the data preprocessing is an inevitable step due to the presence of unwanted artifacts, such as varying image resolution or size, pixel level noise, bright text, and symbols. To address such artifacts, the images are applied to an image mask generated using binary thresholding [25] as given in equation (1). Besides, CXR images may have alterations in the image contrast. To avoid this issue, during the training process, the contrasts of the training images are normalized. Then we denoise the images using filtering. Precisely, the average of the three primary colors red, green, and blue (RGB) image channels is subtracted from each pixel image.

(1) $$\begin{array}{c} \text{Mask}\left(x,y\right)=\left\{\begin{array}{cc} \max_{i}, & i\left(x,y\right)\geq \min_{i},\\ 0, & \text{otherwise.} \end{array}\right. \end{array}$$

Data normalization, which generalizes the effect of different pixel intensities, could be defined in many different forms. Given the pixel intensity noted as PI, the normalized data PI^∗^ is obtained by applying the normalization approach. Here, each pixel ranges from 0 to 255 for each one of the three primary colors. Therefore, the normalization is done through dividing every pixel value by 255. The normalization used in the experiment is done by the maximum and minimum values, which are illustrated in equation (2). Thereafter, it is followed by resizing the images to a fixed size resolution of 224 × 224.

(2) $$\begin{array}{c} P_{I}^{*}=\left(P_{Ij}-{\text{Min}}_{\text{old}}\right)\frac{{\text{Max}}_{\text{new}}-{\text{Min}}_{\text{new}}}{{\text{Max}}_{\text{old}}-{\text{Min}}_{\text{old}}}+{\text{Min}}_{\text{new}},\;j\in \left[0,n\right]. \end{array}$$

#### 3.2.2. Inception-ResNetV2 Model Description

Inception-ResNetV2 model is based on multilayer techniques where every two succeeding layers are linked together by a number of neurons that transform the features in a nonlinearly manner. Network's parameters like weights, biases, activation function, loss model, and optimizer should be carefully allocated. In general, most of DL networks are bidirectional neural networks. The enhanced Inception-ResNetV2 model architecture is presented in Table 3.

**Figure 3:**
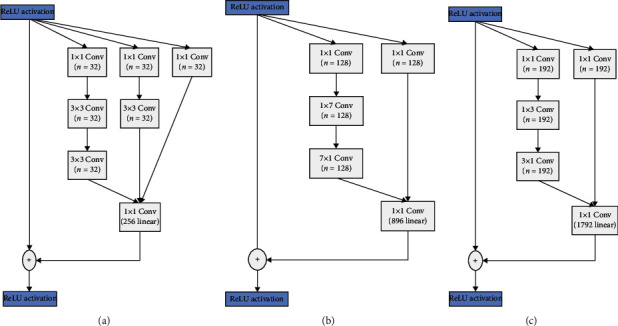
Three types of Inception-ResNetV2 modules from left to right.

**Figure 4:**
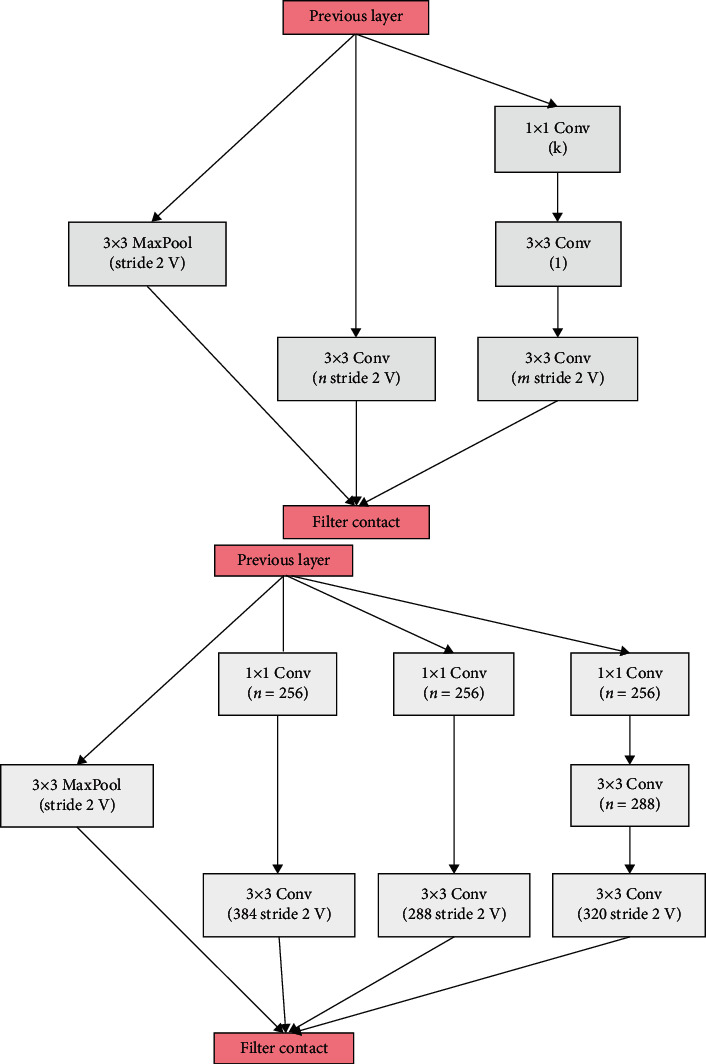
Two schematics of filter contact reduction modules.

Basic parts of Inception-ResNetV2 architecture represented in all layers are established before the fc layer. The Inception-ResNetV2 model contains three basic types of inception modules, namely, Inception-ResNet-A, Inception-ResNet-B, and Inception-ResNet-C as shown in Figure 3. These modules are responsible for both reducing the number of parameters small Conv layers (e.g., 1 × 7, 7 × 1) and generating the discriminatory features. Each module is self-possessed of several Conv and pool layers. Inception-ResNetV2 also contains two types of reduction modules, which are responsible for reducing the image size (see Figure 4). Inception-ResNetV2 model has a default input size 299 × 299; thus, we resized it to 224 × 224 during training.

Figure 3 shows the schematic description for Inception-ResNetV2 network. Inception-ResNetV2 uses the blocks as described in Figure 3. The original Inception-ResNetV2 network output includes 1,000 classes, but only 3 classes are required for our case: COVID-19, viral pneumonia, and normal. Therefore, the output channel number of the last layer (fc) is changed into 3 rather than 1000.

As illustrated in Figure 3, our enhanced version Inception-ResNetV2 comprises a number of convolution layers, followed by 10x Inception-ResNet-A, 20x Inception-ResNet-B, and 10x Inception-ResNet-C, respectively. Thereafter, a 3 × 3 average pooling layer is countered and a Softmax layer comes at the end. To reduce the overfitting, a dropout ratio of 0.5 is utilized following the average pooling. ReLU proceeds for 7 layers of the proposed model from Conv1 through FC7. Meanwhile, the final fc layer has 3 outputs matched to the three classes in the dataset.

For the training phase, the training images are batched in 32 pictures as input to the model. Batch training approach is a beneficial for sinking the storage required for training to be able to fit the whole model in memory and also speed up the training process. Furthermore, the learning rate is set to 0.001, while the dropout rate was set to 0.5. The learning rate is too small to permit the network to find the best global convergence state. The dropout layer role is to inhibit overfitting and help make the trained model more general. In order to alleviate the overfitting, the model has a dropout ratio of 0.5. It requires successive trials to make DL model capable of overcoming both underfitting and overfitting. When the model learns massive details about the training data, it might fall in the issue of overfitting. To prohibit this issue, we utilize early stopping approach, which captures the point where performance on the test dataset starts to go down while performance on the training dataset remains improving. The filters in Inception-ResNetV2 architecture in the various layers could be updated without affecting the accuracy of the trained network. Confidently, we carefully tune the layer sizes to optimize the training speed and balance the computation between the model's subnetworks. Practically, this balance is done through prefetching scheme, which overlaps the preprocessing and model execution of a training step. While the model is executing current training step, the input pipeline is reading the data for the next step. Doing so reduces the step time to the maximum of the training and the time it takes to extract the data.

#### 3.2.3. Discriminative Localization Using Grad-CAM

In many DL applications associated with medical imaging, it is essential to make the results more sensible and explainable. Selvaraju et al. [26] presented a Grad-CAM technique, which provides the explainable visualization of deep learning models and could construct the visual clarification for any DL to learn more about the model during the classification work.

**Figure 5:**
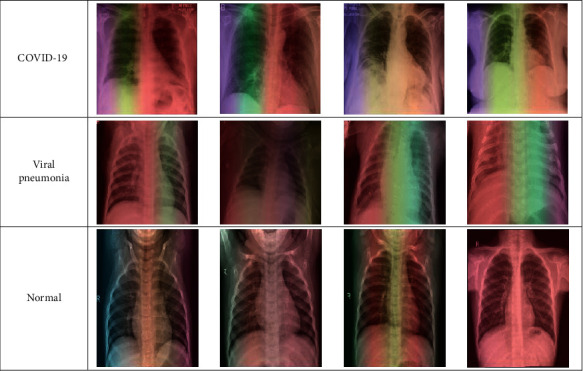
Grad-CAM results for portions of the test CXR images in normal, viral pneumonia, and COVID-19 classes.

As shown in Figure 2, Grad-CAM algorithm is applied to our proposed model through superimposing heat map of CXR dataset images. It produces the class activation mapping by concentrating on the particular portion of COVID-19, viral pneumonia, and normal class. A sample of three class images is examined using Grad-CAM algorithm. In normal X-rays, there is not any kind of opacity that distinguishes normal patients from other patients. As illustrated in Figure 5, there is not any significant region that is localized in normal X-rays. In the case of viral pneumonia, our model has the capability to detect the localized regions with bilateral multifocal ground-glass opacities (GGO) through examining the heatmaps generated. In accordance with [27, 28], there are plenty of similarities between COVID-19 and traditional viral pneumonia, as both of them demonstrate bilateral GGOs along with some patchy consolidations. However, through a deep examination of the heatmap of COVID-19-infected images, it is distinct that the peripheral, diffuse distribution and vascular thickening of such opacities were successfully localized. Therefore, by such localization, the proposed model could assist the clinicians to provide extensive views about the main reasons for the COVID-19 infection. As presented in Figure 5, the dataset images are given as input to the Grad-CAM procedure.

## 4. Implementation and Evaluation

### 4.1. Performance Metrics

To evaluate the proposed model, equations (3)–(6) were employed, namely, accuracy, recall, precision, and *F*1-score. All the following metrics are expressed as percentages. We also used the receiver operating characteristics and the area under the curve.

(3) $$\begin{array}{c} \text{accuracy}=\frac{\text{true positive}+\text{true negative}}{\text{true positive}+\text{true negative}+\text{false positive}+\text{false negative}}, \end{array}$$

(4) $$\begin{array}{c} \text{recall}=\frac{\text{true positive }}{\text{true positive}+\text{false negative}}, \end{array}$$

(5) $$\begin{array}{c} \text{precision}=\frac{\text{true positive}}{\text{true positive}+\text{false positive}}, \end{array}$$

(6) $$\begin{array}{c} F_{1}=2\frac{\text{precision}*\text{recall }}{\text{precision}+\text{recall}}. \end{array}$$

### 4.2. DL Model Performance

In our experiments, the original dataset is randomly divided into 80% for the training phase and 20% for the testing phase while the new one released is divided into 70% for the training phase, 20% for the validation phase, and 10% for the testing phase. The experiments are implemented in python (last release) according to details described in Table 4.

Table 5 shows the average classification results obtained from the 8 DL models for the classification task in the original dataset. It also reports both the training and testing run time in seconds. The maximum measured values are given in italics. As illustrated in the table, Inception-ResNetV2, Xception, VGG16, ResNet50V2, InceptionV3, MobileNetV2, DenseNet121, and ResNet101V2 provide the average accuracy >97%. The highest average accuracy (98.80%) is achieved by Inception-ResNetV2 model. On the contrary, VGG16 and ResNet50 attain the lowest average accuracy (97.60%). Inception-ResNetV2 model gives both the highest *F*1-score (98.86%) and the highest recall (99.11%). MobileNetV2 results in the highest average precision value with 98.67%. However, one of the limitations of our proposed Inception-ResNetV2 model is that it takes a roughly higher training and testing run time compared to other models due to the complex structure of the inside modules.

On the other hand, Table 6 shows the classification results for 3 categories (COVID-19 vs. normal vs. viral pneumonia) obtained from the same 8 DL models for measuring accuracy, *F*1-score, precision, and recall. The highest values are given in italics. As shown in the table, all models achieved an accuracy >97% for COVID-19, normal, and pneumonia classes. Inception-ResNetV2 and DenseNet121 achieved the highest accuracy (98.83%) for COVID-19 category. Inception-ResNetV2 gives both the highest *F*1-score (99.05%) and the highest recall (100%). Besides, Xception and DenseNet121 also attained the highest recall (100%) for COVID-19 category. However, the highest precision (100%) goes for VGG16 and MobileNetV2. It can be noticed that Inception-ResNetV2 has achieved the highest accuracy of 99.83%, the highest F1-score of 98.05%, and the highest recall of 100% for COVID-19 category.

In addition, Table 7 shows the average classification results obtained from the 8 DL models for the classification task in the new release of the dataset (large). As mentioned above, the maximum measured values are given in italics. As shown in the table, our proposed model achieves an average of 97.23%, 96.35%, 96.75%, and 96.00% for accuracy, *F*1-score, precision, and recall, respectively. The highest average accuracy (98.88%) is achieved by DenseNet121 model. Also, it gives the highest *F*1-score (98.18%), highest precision (97.61%), and highest recall (98.78%). On the contrary, Xception attains the lowest average accuracy (50.66%). Then again, Table 8 shows the classification results for 3 categories (COVID-19 vs. normal vs. viral pneumonia) obtained from the same 8 DL models for measuring accuracy, *F*1-score, precision, and recall but according to the large dataset. As shown in the table, our model achieved an accuracy of 98.02% for detecting COVID-19 class. VGG16 achieved the highest accuracy (99.54%) for viral category. It also gives the highest *F*1-score (99%) for normal category while DenseNet121 gives both the highest precision (99.40%) and the highest recall (99.15%) for the same category. In summary, most results obtained from Inception-ResNetV2 in either original dataset or the large dataset are better than those obtained by the other 7 models in terms of accuracy, *F*1-score, precision, and recall.

**Figure 6:**
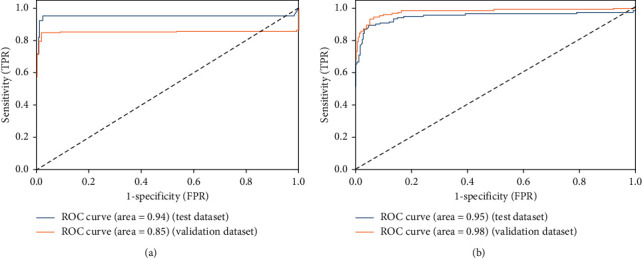
ROC curves for sensitivity (TPR) vs. specificity (FPR) for COVID-19, viral pneumonia, and normal cases.

**Figure 7:**
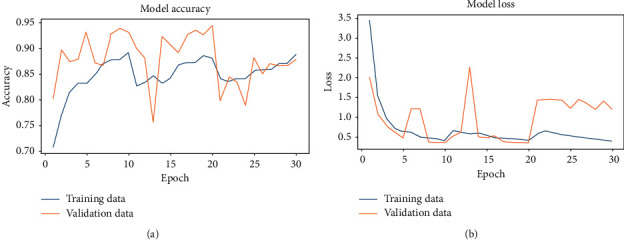
Inception-ResNetV2 model accuracy and model loss for each epoch. (a) Accuracy vs. epoch. (b) Loss vs. epoch.

Figure 6 shows ROC curves in the initial experiment for sensitivity (TPR) vs. specificity (FPR) for normal, viral pneumonia, and COVID-19 disease using the test and validation dataset.  Figure 7 shows both the accuracy and loss for each epoch of training and validation data. It can be noticed that the model accuracy is ranged between 90 and 95% for both training and validation data. Besides, the model loss is dramatically decreased during the epochs 20–30. Moreover, it should be noticed that the training is not stopped early as it is required to complete the whole 5 epochs without any change in the model performance, which has not been achieved here.

### 4.3. Analysis of Inception-ResNetV2 Model

**Figure 8:**
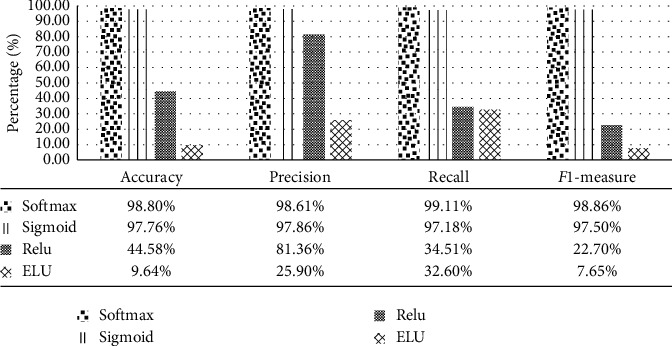
Performance of Inception-ResNetV2 model with different activation functions.

**Figure 9:**
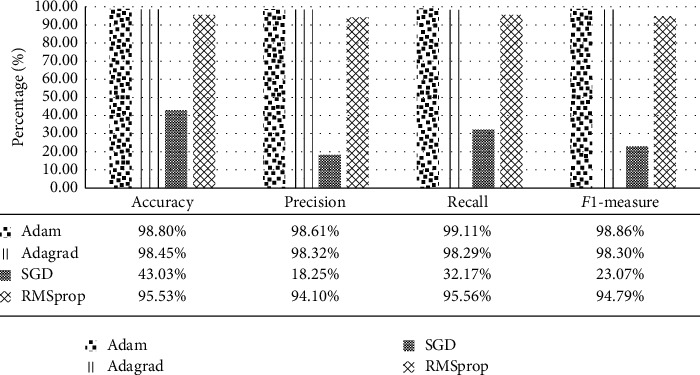
Performance of Inception-ResNetV2 model with different optimizers.

The analysis of our proposed model is carried out with different activation functions, optimizers, and loss model scenarios.  Figure 8 shows the performance of the proposed model with various activation functions: Softmax, Sigmoid, ReLU, and ELU activation functions while fixing the optimizer to Adam and the loss to sparse categorical cross entropy.  Figure 9 shows the performance of the proposed model with Adam, Adagrad, SGD, and RMSprop optimizers while fixing both the activation function and loss of Softmax and sparse categorical cross entropy, respectively. It is noticeable that both Adam and Adagrad optimizers give the best performance compared to other optimizers. SGD records the worst accuracy results due to the demand of a number of hyperparameters and a big number of iterations and it is also sensitive to feature scaling. Besides, ReLU method in the final fc layer forces negative inputs to be zero which ignores many neurons during the training process, thus damaging the capability of the neural net.

**Figure 10:**
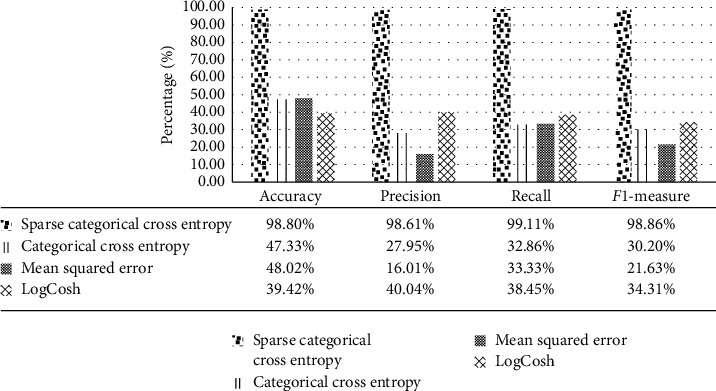
Performance of Inception-ResNetV2 model with different loss functions.

Figure 10 shows performance of the proposed model with sparse categorical cross entropy, categorical cross entropy, mean squared error, and LogCosh loss models while fixing the activation function and optimizer with Softmax and Adam, respectively. Sparse categorical cross-entropy model gives the best accuracy results compared to other loss models. The other three models are dramatically reducing the obtained results.

**Figure 11:**
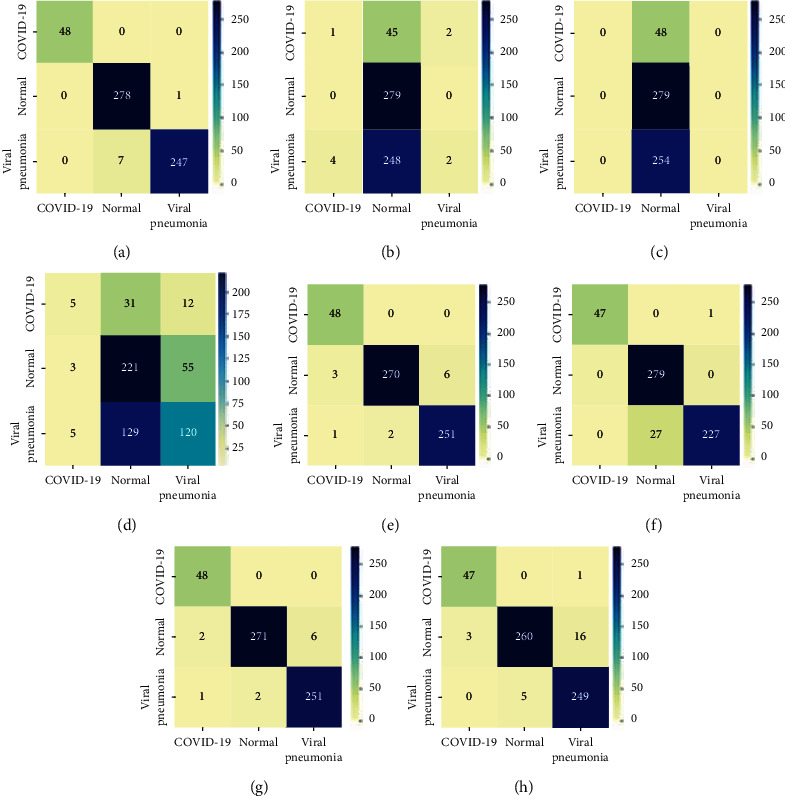
Original dataset 80%–20% confusion matrix of the 8 models for a support of 48, 279, and 254 for COVID-19, normal, and viral pneumonia classes, respectively. (a) Inception-ResNetV2. (b) Xception. (c) VGG16. (d) ResNet50V2. (e) InceptionV3. (f) MobileNetV2. (g) DenseNet121. (h) ResNet101V2.

Figure 11 shows the original dataset 80%–20% confusion matrix of the 8 models for a support of 48, 279, and 254 for COVID-19, normal, and viral pneumonia classes, respectively. It is obvious from Figure 11 that all COVID-19 images (45 images) are accurately classified in Inception-ResNetV2, InceptionV3, and DenseNet121. Specifically, for our proposed model, only 1 normal image out of 279 images is misclassified to viral pneumonia images. Finally, only 7 viral pneumonia images out of 254 images are misclassified to normal class images. Accordingly, it is great that none of COVID-19 images are misclassified to the other disease categories. Similarly, only seven viral pneumonia images were misclassified to normal class. However, it is also less severe than misclassifying COVID-19 images. Compared to both InceptionV3 model and DenseNet121 model, it is noticeable that our model is not confusing in COVID-19 images; rather, it is roughly confused between normal images and viral pneumonia images.

**Figure 12:**
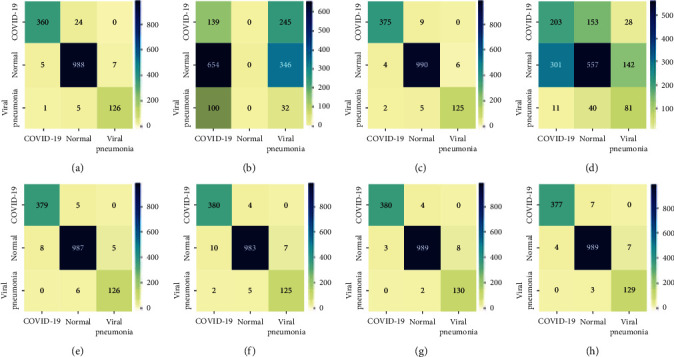
Large dataset (new release) 70%-20%-10% confusion matrix of the 8 models for a support of 384, 1000, and 132 for COVID-19, normal, and viral classes, respectively. (a) Inception-ResNetV2. (b) Xception. (c) VGG16. (d) ResNet50V2. (e) InceptionV3. (f) MobileNetV2. (g) DenseNet121. (h) ResNet101V2.

Further, Figure 12 shows the large dataset 70%-20%-10% confusion matrix of the 8 models for a support of 384, 1000, and 132 for COVID-19, normal, and viral classes, respectively. Specifically, for our proposed model, 24 COVID-19 images out of 384 are misclassified to normal class. Meanwhile, 9 normal images out of 1000 are misclassified where 3 images are classified as COVID-19 and 6 images as viral. Finally, only 6 viral pneumonia images out of 132 images are misclassified where an image is classified as COVID-19 and 5 images as normal. Generally, according to the large dataset, DenseNet121 achieves the best results compared to others. However, our proposed model achieves an average 97.23%, 96.35%, 96.75%, and 96.00% for accuracy, *F*1-score, precision, and recall, respectively.

### 4.4. Comparison with State-of-the-Art Methods

Compared to state-of-the-art methods reported in Table 9, the obtained results revealed the superiority of our proposed model in the matter of accuracy for 3-class classification task. It gets remarkable average accuracy results (98.80) due to concentration on the residual connection rather than filter connection via split and merge as previously shown in Figures 3 and 4. Moreover, it supports dimension reduction in a way that promotes faster learning. For CXR images, these results of detecting COVID-19 are considered promising and encouraging. It could significantly assist radiologists to avoid heaviness of hospitals and medical systems. Our proposed model is effective enough to support radiologists in the diagnosis of COVID-19 as it is capable of classifying COVID-19 successfully with an accuracy reaching 99.11%.

## 5. Conclusions and Future Work

In this paper, an ensemble of deep learning models is used in the study to classify patients affected by COVID-19 using CXR images. Using the transfer learning approach, the models have been trained for dataset of images of COVID-19 dataset. The study is more oriented to Inception-ResNetV2 model due to its high metrics. Compared to state-of-the-art methods, our base model named Inception-ResNetV2 achieved an average accuracy of 99.83% for detecting COVID-19 and an average accuracy of 98.80% for three-class classification, which confirms superiority in classifying COVID-19 cases. Additionally, our proposed model could be assisting radiologists in the diagnosis of COVID-19 infection quickly. In the future, we will work to develop our proposed work to detect the severity of COVID-19 infection cases via a patient image bank. Moreover, the computational complexity of our proposed model will be considered and criticized, and the model will be also validated using CT images coming from different sources.

## Figures and Tables

**Table 1:** The distribution of training and testing sets used in the original dataset.

Category	No. of images	Training	Testing
COVID-19	219	171	48
Viral pneumonia	1345	1066	279
Normal	1341	1087	254

**Table 2:** The distribution of training, validation, and testing sets used in the new release of the dataset.

Category	No. of images	Training	Validation	Testing
COVID-19	3616	2464	768	384
Viral pneumonia	10192	949	264	132
Normal	1345	7192	2000	1000

**Table 3:** Inception-ResNetV2 model architecture.

Layer	Patch size	Input size
Conv	3 × 3	224 × 224 × 3
Conv	3 × 3	111 × 111 × 32
Filter contact	3 × 3 pool + 3 × 3 conv	109 × 109 × 64
Filter contact	1 × 1 conv, 3 × 3 conv+ 1 × 1 conv, 7 × 1 conv, 1 × 7 conv, 3 × 3 conv	54 × 54 × 160
Filter contact	3 × 3 conv + max pool	52 × 52 × 128
Inception-ResNet-A × 10	—	26 × 26 × 256
Reduction-A	—	26 × 26 × 256
Inception-ResNet-B × 20	—	13 × 13 × 768
Reduction-B	—	13 × 13 × 768
Inception-ResNet-C × 10	—	6 × 6 × 1534
Average pooling	6 × 6	6 × 6 × 1534
Dropout	Keep = 0.5	1 × 1 × 1534
Fc	1534 × 1000	1534
Fc	1000 × 3	1000
Softmax	Classifier (3 classes)	500

**Table 4:** Machine description.

CPU model name	Intel (R) Xeon (R) CPU @ 2.30 GHz
CPU cores	16
RAM	13 gigabytes
GPU card	NVIDIA Quadro K6000 16 GB
Operating system (OS)	Linux 0e22a0d3b32a 4.9.0-5-amd 64 #1

**Table 5:** Average classification results for the classification task of the original dataset.

Model	Accuracy (%)	*F*1-score (%)	Precision (%)	Recall (%)	Training time (s)	Testing time (s)
Inception-ResNetV2	*98.80*	*98.86*	98.61	*99.11*	*1138*	*4.10*
Xception	98.30	98.45	98.15	98.78	1051	2.13
VGG16	97.60	97.30	98.29	96.40	912	1.85
ResNet50V2	97.60	97.65	97.08	98.28	890	2.28
InceptionV3	97.90	98.20	97.90	98.51	894	2.16
MobileNetV2	98.10	97.67	*98.67*	96.77	881	1.74
DenseNet121	98.30	98.45	98.15	98.78	940	1.91
ResNet101V2	97.40	97.19	97.50	96.90	1006	2.55

**Table 6:** Model classification results for 3-category (COVID-19 vs. normal vs. viral pneumonia) classification task for the original dataset.

Model	Category	Accuracy (%)	*F*1-score (%)	Precision (%)	Recall (%)
Inception-ResNetV2	COVID-19	*99.83*	*99.05*	98.11	*100.00*
	Normal	98.80	98.62	98.81	98.43
	Viral pneumonia	98.97	98.91	98.91	98.91

Xception	COVID-19	99.83	98.90	97.83	*100.00*
	Normal	98.28	98.16	99.63	96.74
	Viral pneumonia	98.45	98.29	97.00	99.62

VGG16	COVID-19	99.48	96.55	*100.00*	93.33
	Normal	97.59	97.48	96.79	98.19
	Viral pneumonia	98.11	97.88	98.07	97.69

ResNet50V2	COVID-19	99.66	97.83	95.75	100.00
	Normal	97.76	97.62	98.89	96.38
	Viral pneumonia	97.76	97.52	96.60	98.46

InceptionV3	COVID-19	99.83	98.90	97.83	100.00
	Normal	98.11	98.00	98.54	97.46
	Viral pneumonia	97.93	97.70	97.33	98.08

MobileNetV2	COVID-19	99.48	96.55	*100.00*	93.33
	Normal	98.28	98.21	97.16	99.28
	Viral pneumonia	98.45	98.26	98.83	97.69

DenseNet121	COVID-19	*99.83*	98.90	97.83	*100.00*
	Normal	98.28	98.16	99.63	96.74
	Viral pneumonia	98.45	98.29	97.00	99.62

ResNet101V2	COVID-19	99.48	96.63	97.73	95.56
	Normal	97.93	97.83	97.83	97.83
	Viral pneumonia	97.42	97.12	96.94	97.31

**Table 7:** Average classification results for the classification task of the new release of the dataset.

Model	Accuracy (%)	*F*1-score (%)	Precision (%)	Recall (%)	Training time (s)	Testing time (s)
Inception-ResNetV2	97.23	96.35	96.75	96.00	*3449*	*10.78*
Xception	50.66	32.02	29.27	30.50	2942	5.74
VGG16	98.29	97.30	97.48	97.12	1833	4.85
ResNet50V2	55.48	50.37	48.65	56.64	1496	5.85
InceptionV3	98.42	97.64	97.67	97.62	1587	5.70
MobileNetV2	98.15	97.11	96.91	97.32	1406	4.64
DenseNet121	*98.88*	*98.18*	*97.61*	*98.78*	1938	4.89
ResNet101V2	98.62	97.93	97.60	98.27	2441	6.59

**Table 8:** Model classification results for 3 categories (COVID-19 vs. normal vs. viral pneumonia) classification task for the new release of the dataset.

Model	Category	Accuracy (%)	*F*1-score (%)	Precision (%)	Recall (%)
Inception-ResNetV2	COVID-19	98.02	96.00	98.36	93.75
	Normal	97.36	97.97	97.15	98.80
	Viral pneumonia	98.94	95.09	94.74	95.46

Xception	COVID-19	44.85	36.20	15.57	21.77
	Normal	50.73	64.16	63.53	64.80
	Viral pneumonia	89.38	24.24	5.14	8.48

VGG16	COVID-19	99.27	97.66	98.43	98.04
	Normal	98.81	*99.00*	98.61	98.80
	Viral pneumonia	*99.54*	94.70	95.42	95.06

ResNet50V2	COVID-19	57.92	52.87	39.42	45.16
	Normal	52.24	55.70	74.27	63.66
	Viral pneumonia	91.16	61.36	32.27	42.30

InceptionV3	COVID-19	98.88	98.70	97.93	98.31
	Normal	98.35	98.70	98.90	98.80
	Viral pneumonia	99.21	95.46	96.18	95.82

MobileNetV2	COVID-19	98.68	98.96	96.94	97.94
	Normal	98.15	98.30	99.09	98.70
	Viral pneumonia	98.15	94.70	94.70	94.70

DenseNet121	COVID-19	99.34	98.96	99.22	99.09
	Normal	98.48	98.90	*99.40*	*99.15*
	Viral pneumonia	99.14	98.49	94.20	96.30

ResNet101V2	COVID-19	99.21	98.18	98.95	98.56
	Normal	98.68	98.90	99.00	98.95
	Viral pneumonia	99.47	97.73	94.85	96.27

**Table 9:** The results obtained compared to state-of-the-art methods.

Reference	Utilized models	Highest achievement
Wang et al. [12]	COVID-Net	Accuracy: 92.4% for 2 classes
		83.5% for 4 classes
Hemdan et al. [19]	COVIDX-Net	*F*1-score: 0.89 for normal
		0.91 for COVID-19
Sethy and Behera [20]	ResNet50 and SVM classifier	Accuracy: 95.38%
Ozturk et al. [29]	Dark COVID-Net	Accuracy: 87.02% for 3 classes
Apostolopoulos and Mpesiana [30]	VGG-19	Accuracy: 93.48% for 3 classes
Khan et al. [18]	CoroNet	Accuracy: 89.6% for 4 classes
		95% for 3 classes
Xu et al. [16]	ResNet	Accuracy: 86.7%
Li et al. [31]	COVNet	Specificity: 96%
		Sensitivity: 90%
		AUC: 96%
Song et al. [15]	DeepPneumonia	Accuracy: 92.4% for 2 classes
Ghoshal and Tucker [32]	Bayesian CNN	Accuracy: 92.90%
Zhang et al. [2]	Deep CNN based on backbone network	Specificity: 70.7%
		Sensitivity: 96.0%
		AUC: 95.2%
Ouchicha et al. [21]	CVDNet	Accuracy: 97.20% for 2 classes
		96.69 for 3 classes (COVID-19 vs. normal vs. viral pneumonia)
Our proposed	Enhanced Inception-ResNetV2	Accuracy: 98.80% (average accuracy) and 99.20 for 3 classes (COVID-19 vs. normal vs. viral pneumonia)
		*F*1-score: 98.86%
		Precision: 98.61%
		Recall: 99.11
		AUC: 97.2%

## Data Availability

Publicly available datasets were analyzed in this study. These datasets can be found at COVID-19 Radiography Dataset, available online at https://www.kaggle.com/tawsifurrahman/covid19-radiography-database.
